# Inhibition of growth of *Zymomonas mobilis* by model compounds found in lignocellulosic hydrolysates

**DOI:** 10.1186/1754-6834-6-99

**Published:** 2013-07-09

**Authors:** Mary Ann Franden, Heidi M Pilath, Ali Mohagheghi, Philip T Pienkos, Min Zhang

**Affiliations:** 1 National Bioenergy Center, National Renewable Energy Laboratory, 15013 Denver West Parkway, Golden, CO 80401, USA

**Keywords:** *Zymomonas mobilis*, High-throughput screening, Cell growth assay, Bioscreen C, Inhibitor, Hydrolysate, Lignocellulosic biomass, Ethanol

## Abstract

**Background:**

During the pretreatment of biomass feedstocks and subsequent conditioning prior to saccharification, many toxic compounds are produced or introduced which inhibit microbial growth and in many cases, production of ethanol. An understanding of the toxic effects of compounds found in hydrolysate is critical to improving sugar utilization and ethanol yields in the fermentation process. In this study, we established a useful tool for surveying hydrolysate toxicity by measuring growth rates in the presence of toxic compounds, and examined the effects of selected model inhibitors of aldehydes, organic and inorganic acids (along with various cations), and alcohols on growth of *Zymomonas mobilis* 8b (a ZM4 derivative) using glucose or xylose as the carbon source.

**Results:**

Toxicity strongly correlated to hydrophobicity in *Z. mobilis,* which has been observed in *Escherichia coli* and *Saccharomyces cerevisiae* for aldehydes and with some exceptions, organic acids. We observed *Z. mobilis* 8b to be more tolerant to organic acids than previously reported, although the carbon source and growth conditions play a role in tolerance. Growth in xylose was profoundly inhibited by monocarboxylic organic acids compared to growth in glucose, whereas dicarboxylic acids demonstrated little or no effects on growth rate in either substrate. Furthermore, cations can be ranked in order of their toxicity, Ca^++^ > > Na^+^ > NH4^+^ > K^+^. HMF (5-hydroxymethylfurfural), furfural and acetate, which were observed to contribute to inhibition of *Z. mobilis* growth in dilute acid pretreated corn stover hydrolysate, do not interact in a synergistic manner in combination. We provide further evidence that *Z. mobilis* 8b is capable of converting the aldehydes furfural, vanillin, 4-hydroxybenzaldehyde and to some extent syringaldehyde to their alcohol forms (furfuryl, vanillyl, 4-hydroxybenzyl and syringyl alcohol) during fermentation.

**Conclusions:**

Several key findings in this report provide a mechanism for predicting toxic contributions of inhibitory components of hydrolysate and provide guidance for potential process development, along with potential future strain improvement and tolerance strategies.

## Background

From a process economic consideration, two of the biggest challenges for a cost competitive cellulosic ethanol operation involve achieving high sugar yields from the hydrolysis of cellulose and hemicellulose in the pretreatment and enzymatic saccharification steps and subsequent fermentation of those sugars to ethanol at high yields. Pretreatment processes are designed to break down the cellulose, hemicellulose and lignin matrix, releasing monosaccharides and providing access of polysaccharides for enzymatic conversion and they often result in the introduction of toxic compounds which are also inhibitory to microbial fermentations [1-6], and so the two challenges have appeared to be mutually exclusive.

Inhibitors fall into several categories: furans, phenols, carboxylic and inorganic acids, aldehydes and alcohols [6-8]. Organic acids (primarily acetic acid) are released from the deacetylation of xylan and lignin breakdown during pretreatment [1,9]. The furans, HMF and furfural, result from the degradation of hexose and pentoses, respectively. Other degradation products include formic, 2-furoic, and levulinic acids. Phenols identified in lignocellulosics include acids, alcohols, aldehydes and ketones [7]. The extent of inhibitor formation is often a function of pretreatment severity, a combined factor that includes reaction time, temperature, and catalyst concentration. More severe pretreatment conditions generally produce pretreated solids that are easily saccharified by enzymes, but such conditions often cause greater sugar degradation losses and require costly detoxification steps, often referred to as conditioning.

Most of the published work focusing on mitigating the negative influence of inhibitors in hydrolysates has relied on chemical, physical or biological methods for their removal or abatement. These methods include overliming, ion-exchange chromatography, the use of polymeric sorbents, or treatments with enzymes or microorganisms [10]. A more systematic approach to this problem would involve the identification and potency of inhibitors, understanding the basis of their formation, and identifying alternative pretreatment conditions and/or techniques to prevent their production. The efficacy of conditioning treatments can be determined by benchmarking the fermentation of the conditioned hydrolysate to a control, consisting of a reference medium supplemented with pure sugars. While these assessments provide guidance, they provide little information about the type and quantity of the inhibitors removed and the potential synergistic effects between various inhibitory components that may be present.

Because of the many variables reported for hydrolysate toxicity measurements, such as fermentation organisms, inoculum levels, fermentation conditions, biomass feedstocks, and pretreatment conditions [11-14], it is difficult to interpret published data and properly evaluate the toxic nature of hydrolysates. The contribution of individual compounds to the overall toxic level and the underlying toxicity mechanisms are not well understood at the level required for optimization of the pretreatment conditions or effectively engineering tolerant strains. Some work has been conducted analyzing the inhibitory effects of model compounds in *Z. mobilis*, *E. coli*, *S. cerevisiae*, etc. [4,5,13,15-18]. For example, the recombinant strain *E. coli* LY01 was tested for growth and ethanol production inhibitions by a wide range of aldehydes, acids, and alcohols [16-18]. In these works, inhibitory activity was closely related to hydrophobicity. Aldehydes tended to be more toxic than acids, which were more toxic than alcohols.

Little is known of the toxic effects of inhibitory compounds on *Z. mobilis* growth and fermentation, although some preliminary studies have evaluated the effects of furans, aldehydes in glucose with a wild-type *Z. mobilis* ATCC 10988 and with a recombinant plasmid-bearing strain *Z. mobilis* CP4/pZB5 [15]. *Z. mobilis* ZM4 has been shown to be more robust in its enhanced sugar utilization rates and tolerance to ethanol [14,19]. *Z. mobilis 8b*, a recombinant xylose utilizing strain derived from ZM4 [20], was shown to have improved tolerance to acetic acid [21]. Nevertheless, the toxic effects on *Z. mobilis* cell growth by hydrolysates or inhibitors have not been investigated systematically. To this end, we developed a quantitative, high-throughput biological growth assay using an automated turbidometer to obtain detailed inhibitory kinetic data for individual compounds [22]. Growth is widely used to evaluate the toxicity of various inhibitor compounds on microbial fermentation and is associated with ethanol production by recombinant *S. cerevisiae*, *E. coli* and *Z. mobilis*. Inhibition of cell growth has been shown to be strongly correlated with inhibition of ethanol production for many inhibitory compounds [4,5,16,17,23,24].

In this study, we examined model inhibitor compounds that include five aldehydes, fourteen organic acids and two alcohols, for their effect on growth rates as well as final cell mass using either glucose or xylose as the substrate. Since, inorganic salts can also be introduced during the acid or alkali pretreatment processes and subsequent neutralization or conditioning steps preceding saccharification, we also compared four cations and four inorganic anions for their inhibitory potential as well. We further investigated synergistic potentials of HMF, furfural, acetate and formate. Furfural was found to inhibit growth and fermentation synergistically with phenols in *E. coli*[16-18] and with acetic acid in *S. cerevisiae*[4].

## Results and discussion

We previously reported a method for examining growth inhibition with individual compounds as well as hydrolysate using a high throughput assay and described the inhibition on growth and final cell densities of *Z. mobilis* by four compounds: acetate, ethanol, HMF and furfural [22]. We continued to use this method for a more detailed analysis of the effect of compounds that are either produced or introduced as a result of the pretreatment and conditioning steps. This was done in order to determine the relative toxicity among the inhibitors and provide guidance for improvements in both biomass processing steps or in the robustness of the ethanologen.

### Effect of inhibitors on growth of *Z. mobilis* in glucose

**Figure 1:**
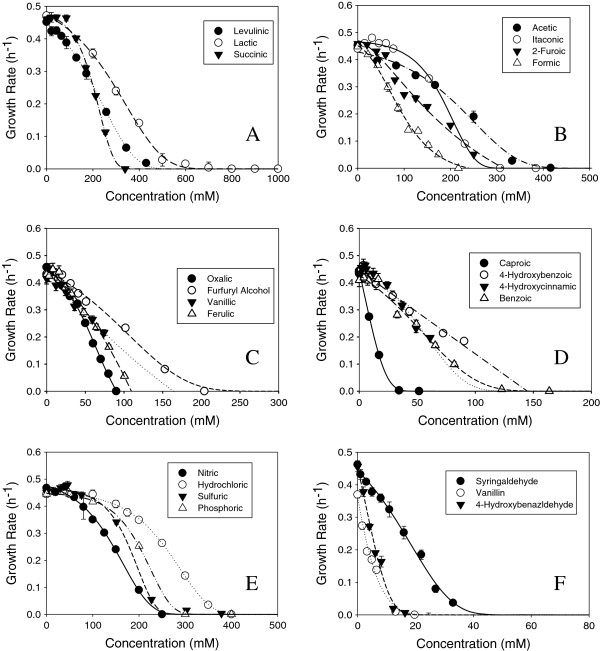
**Growth rates for *Z. mobilis *8b in RMG with increasing concentrations of inhibitor. A)** ammonium levulinate, ammonium lactate, ammonium succinate; **B)** ammonium acetate, ammonium itaconate, ammonium 2-furoate and ammonium formate; **C)** potassium oxalate, furfuryl alcohol, ammonium vanillate and ammonium ferulate; **D)** ammonium caproate, ammonium 4-hydroxybenzoate, ammonium 4-hydroxycinnamate and ammonium benzoate; **E)** ammonium nitrate, ammonium hydrochlorate, ammonium sulfate and ammonium phosphate; **F)** syringaldehyde, vanilin,4-hydroxybenzaldehyde.

We used the Bioscreen C to determine growth rates for *Z. mobilis* 8b grown in the compounds listed in Table 1. All of these compounds have been identified, with the exception of oxalic acid, in dilute acid corn stover hydrolysates. Growth curves from cultures using glucose as the sole carbon source are shown in Figure 1 for each inhibitor concentration tested. Maximum growth rates, calculated after the cells doubled at least once within a 24 hour period, are plotted against the inhibitor concen-tration. The ammonium cation was used for each acid presented in Figure 1, with the exception of oxalic acid. Ammonium oxalate is insoluble in water above 50 mM at 30°C, therefore the potassium form was tested. Panels A, B, C and D display growth rate profiles for organic inhibitors and furfuryl alcohol are ranging from the least toxic organic acids in panel A to the most toxic in panel D to allow for better visualization of the data. Panels E and F show inhibition profiles of growth rate for *Z. mobilis* 8b grown in the presence of inorganic acids and three aldehydes, respectively. The growth curve profiles for HMF, furfural, and ethanol have already been reported [22]. Not surprisingly, ethanol was the least toxic compound studied. *Z. mobilis* is known for its high tolerance to ethanol. The parent strain, ZM4, has been shown to exhibit a similar specific growth rate when exposed to ethanol under continuous culture experiments [19].

**Table 1:** **Hydrophobicity values (logP**_**octanol/water **_**coefficients), millimolar inhibitory concentrations (IC**_**25**_**, IC**_**50**_**, IC**_**75 **_**and IC**_**100**_**) for growth rate inhibitions by 25%, 50%, 75% and 100%, respectively in glucose and xylose**

		**RMG**				**RMX**			
**Compound**	**Hydrophobicity (logP**_**octanol/water**_**)**	**IC**_**25**_	**IC**_**50**_	**IC**_**75**_	**IC**_**100**_	**IC**_**25**_	**IC**_**50**_	**IC**_**75**_	**IC**_**100**_
**Organic acids**^**9**^									
Oxalic acid	-2.20 ^1^	33	53	71	90	29	59	86	105
Lactic acid	-0.60 ^3^	210	315	415	600	100	170	265	400
Succinic acid	-0.59 ^2^	165	210	260	340	165	195	260	340
Formic acid	-0.54 ^2^	50	85	130	240	14	23	35	65
Levulinc acid	-0.49 ^5^	130	220	300	475	35	50	65	90
Acetic acid	-0.17 ^2^	140	210	280	360	25	50	70	110
2-Furoic acid	0.64-0.73 ^3^	85	145	215	300	30	60	100	180
Itaconic acid	0.71 ^1^	150	185	220	320	185	220	250	385
Vanillic acid ^10^	1.43 ^4^	33	70	**105**	**145**	27	40	55	74
Ferulic acid	1.51 ^6^	40	65	90	120	35	55	90	120
4-Hydroxybenzoic acid ^10^	1.58 ^6^	35	75	**105**	**145**	25	40	55	90
4-Hydroxycinnamic acid ^11^ (ρ-Coumaric acid)	1.79 ^7^	30	55	**80**	**105**	21	34	47	61
Benzoic acid	1.87 ^2^	25	55	75	125	8	9	18	33
Caproic (Hexanoic) acid	1.92 ^2^	7	12	18	34	3	5	9	17
**Inorganic acids**^**9**^									
Hydrochloric acid		215	260	305	400	250	305	355	400
Sulfuric acid		150	180	205	300	230	255	275	300
Phosphoric acid		190	240	285	330	340	375	400	460
Nitric acid		115	155	190	250	105	150	185	210
**Aldehydes**									
HMF	-0.37 ^8^	10*	22*	37*	63*	12	26	42	63
Furfural	0.41 ^2^	8*	17*	27*	52*	9	16	26	42
Syringaldehyde ^11^	0.99 ^8^	11	18	26	40	5	10	14	28
Vanillin	1.21 ^2^	2	4	9	20	1	3	5	13
4-Hydroxybenzaldehyde	1.35 ^5^	2	5	10	25	3	7	12	25
**Alcohols**									
Furfuryl alcohol	0.28 ^2^	60	100	140	220	60	100	140	205
Ethanol	-0.24 ^2^	910*	1350*	1735*	2170*	950	1260	1600	2170

^1^ Estimated Values from Yaws’ Handbook of Thermodynamic and Physical Properties of Chemical Compounds © 2003 Knovel, Table: Solubility in Water and Octanol-Water Partition Coefficient.

^2^ Measured Values from Yaws’ Handbook of Thermodynamic and Physical Properties of Chemical Compounds © 2003 Knovel, Table: Solubility in Water and Octanol-Water Partition Coefficient.

^3^ Handbook of Environmental Data on Organic Chemicals (4th Edition) © 2001 John Wiley & Sons; Table: Physical and Environmental Data on Organic Chemicals (2-Furoic Acid is calculated).

^4^ Perry’s Chemical Engineers’ Handbook (7th Edition) © 1997 McGraw-HillChemicals; Table: Safety Properties of Common Solvents.

^5^ Chemical Properties Handbook © 1999 McGraw-Hill; Table:

^6^ Exploring QSAR,Hydrophobic, Electronic, and Steric Constants Hansch C, Leo A, and Hoekman D 1995 Amer. Chem. Soc., Washington DC.

^7^ Octanol-Water Partition Coefficients: Fundamentals and Physical Chemistry. Sangster J 1994 Wiley, New York.

^8^ Partition coefficients calculated from Biobyte, Inc. (Clarement, CA), cited [16-18].

^9^ Inhibitory concentrations (IC) obtained for the NH4^+^ cation of the acids, except for oxalic acid in which case, K^+^ was used).

^10^ Inhibitory concentrations in bold were extrapolated from the inhibition curve.

^11^ Indicated compounds were dissolved in DMSO (< 5% (v/v)) because of low solubilities.

* Reported [22].

Each compound elicited a distinct growth rate inhibition profile which is either sigmoidal or hyperbolic in shape. Lactic acid, which is sometimes introduced by contamination of lactic acid bacteria in biomass fermentations, is tolerated by *Z. mobilis* up to 500-600 mM (Figure 1A). Levulinic, succinic, acetic, itaconic and 2-furoic share similar minimum inhibitory concentrations (MIC) of 300-475 mM (Figure 1A and B). MIC is defined as the lowest concentration of inhibitor that completely inhibits growth within a 24 hour period. However, at concentrations below 100 mM, succinic and itaconic were less inhibitory than acetic, levulinic and 2-furoic acids. Due to low solubilities of vanillic, 4-hydroxybenzoic, and 4-hydroxycinnamic (ρ-coumaric) acids, concentrations causing more than 50% inhibition were not tested; therefore MICs were extrapolated from the inhibition curves (Figure 1C and D). Caproic (hexanoic) acid was most inhibitory at concentrations as low as 34 mM, followed by 4-hydroxycinnamic, oxalic, 4-hydroxybenzoic, vanilic, ferulic, formic, 2-furoic, itaconic, succinic, acetic, levulinic and lactic acid. Oxalic acid is not recognized as a component of lignocellulosic hydrolysate but was included in this study because it has been used or considered for some pretreatment processes [25].

Of the inorganic salts studied, ammonium chloride is the least toxic and ammonium nitrate is the most toxic. Ammonium phosphate is slightly less toxic than ammonium sulfate (Figure 1E). Sulfuric and phosphoric acid are divalent and trivalent, respectively, and would contain additional ammonium ions increasing its ionic strength as well as the osmolarity of the medium. Sulfuric acid is often used in dilute acid pretreatment processes and ammonium sulfate at concentrations above 300 mM (~40 g/L) completely inhibits *Z. mobilis* growth.

The aldehydes were far more toxic than the organic acids used in this study with the exception of caproic acid. The most toxic aldehyde was vanillin, followed by 4-hydroxybenzaldehyde, syringaldehyde, furfural and HMF (Figure 1F). Furfuryl alcohol was examined in this study as it has been shown to be a product of furfural conversion in *Z. mobilis* (Additional file 1: Figure S1A and Additional file 2: Figure S2C) and is approximately one fourth as inhibitory as furfural (Figure 1C).

### Relationship of compound hydrophobicity to growth of *Z. mobilis*

**Figure 2:**
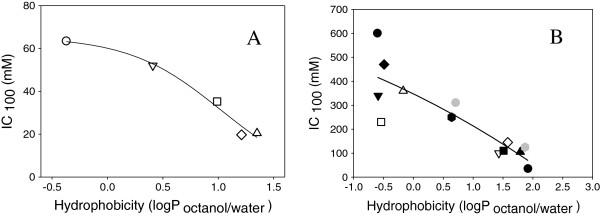
**Hydrophobicity as LogP**_**octanol/water **_**partition coefficients plotted with the minimal inhibitory concentrations (IC**_**100**_**) of aldehydes. A)** HMF (○), furfural (∇), syringaldehyde (□), vanillin (◊), and 4-hydroxybenzaldehyde (∆); and organic acid compounds **B)** ammonium lactate (●), ammonium levulinate (♦), ammonium succinate (▼), ammonium formate (□), ammonium acetate (∆), ammonium 2-furoate (), ammonium itaconate (gray octagon), ammonium vanillate (∇), ammonium ferulate (■), ammonium 4-hydroxybenzoate (◊), ammonium 4-hydroxycinnamate (▲), ammonium benzoate (gray hexagon), and ammonium caproate (●).

Inhibitory millimolar concentrations of compounds at 25% (IC_25_), 50% (IC_50_), 75% (IC_75_) and 100% (IC_100_ or MIC) were estimated from the growth rate profile and are presented along with octanol/water partition coefficients (logP _octanol/water_) in Table 1. The relationship between MIC and logP is also shown graphically in Figure 2. The toxicity ranking for aldehydes tested followed the same trend as described for *E. coli* which also correlated to their logP values [16,17]. This is also in agreement with observations that compounds containing methoxy substituents ortho to the phenol hydroxyl group decrease the toxicity of phenols towards *S. cerevisiae*[2,6,7,13,26].

Hydrophilic organic acids tended to be less inhibitory than the hydrophobic ones, though some compounds with negative logP values did not follow this trend. Although oxalic acid, a dicarboxylic acid with pK_a_ values of 1.3 and 4.3) is very hydrophilic, it was extremely toxic to *Z. mobilis*. Oxalic acid has been used as a catalyst for pretreatment of biomass [25]. It is a very strong acid and forms insoluble precipitates with many metal ions, including magnesium, which is essential for many important enzymes (pyrophosphatase, pyruvate kinase, enolase, membrane ATPase, and others) [27-30]. Lactic, levulinic, acetic, succinic and formic acids have similar partition coefficients yet vary significantly in their inhibitory level. Formic acid, a degradation product of HMF and furfural, is also very toxic to *Z. mobilis* and has also been observed to be toxic to *E. coli*. [16]. It has been proposed that its higher toxicity may be a result of its high permeability through the cell membrane [31,32]. Compounds with lower hydrophobicity (*i.e.* when logP_(octanol/water)_ < 1) generally had lower toxicity. Other less inhibitory organic acids included lactic acid, followed by levulinic and acetic acid.

### Relationship of growth rates to final cell densities

Growth rates for selected compounds at varied concentrations are plotted with the relative growth yield taken after 24 hours (Additional file 3: Figure S3). The relative growth yield is the ratio of the maximum cell density +/- inhibitor, correcting values for non-linear response [22]. Results for growth rates and final cell densities for acetate, HMF, furfural and ethanol were reported previously [22]. All of the compounds tested caused a decrease in final cell densities, but displayed different inhibitory profiles. Only representative compounds are shown (Additional file 3: Figure S3). Formic (Additional file 3: Figure S3A), caproic, and benzoic acid as well as syringaldehyde caused significant reductions in final cell mass relative to the reduction in growth rates. This is similar to the pattern observed with acetic acid [22]. For most of the other acids and alcohols as well as 4-hydroxybenzaldehyde and vanillin, final cell density was reduced to a level consistent with the decrease in growth rates for increasing concentrations of inhibitor as shown for itaconic acid in Additional file 3: Figure S3B. On the other hand, whereas growth rates in HMF were reduced, cell mass at the end of fermentation (24 hours) was not affected until concentrations exceeded 12 mM (1.5 g/L) [22] and then the reduction in growth rates for increasing inhibitor concentrations exceeded the reduction in cell density. This signified that the compounds did not affect the stoichiometry of the conversion of substrates into cellular material, only the conversion rate. Similarly, growth rates in furfural were more affected than final cell density [22]. Other compounds showing a similar pattern include: 4-hydroxycinnamic acid, 4-hydroxybenzoic acid (Additional file 3: Figure S3C), and vanillic acid and possibly ferulic acid.

For the inorganic acids, relative final cell densities were proportional to growth rates, with the possible exception of sulfate, which did not reduce final cell mass even when growth rates were reduced (Additional file 3: Figure S3D).

### Growth inhibition and carbon source

**Figure 3:**
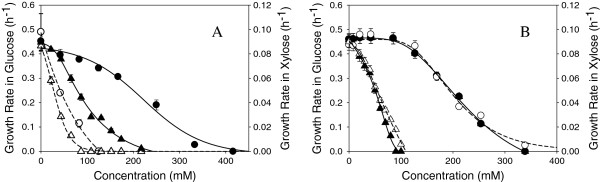
**Growth rates for *Z. mobilis *8b grown in glucose (closed symbols) or xylose (open symbols) in A) ammonium acetate (●) or ammonium formate (▲); B) ammonium succinate (●) or ammonium ferulate (▲).**

We also examined the different effect of the inhibitors on cells grown in the presence of either glucose or xylose. Estimated millimolar concentrations for compounds causing 25%, 50%, 75% and 100% inhibitions are given in Table 1 and inhibitory profiles of several compounds on both glucose and xylose growth are given in Figure 3. *Z. mobilis* 8b was particularly sensitive to formic and acetic acid (Figure 3) as well as benzoic, levulinic, caproic, lactic and furoic acids using xylose as the sole carbon source since inhibitory concentrations were nearly 2-4 fold lower in xylose compared with levels required to inhibit growth in glucose. *Z. mobilis* 8b was slightly more sensitive to growth on xylose in 4-hydroxybenzoic acid, 4-hydroxycinnamic and vanillic acid. Similarly, syringaldehyde, and vanillin inhibited growth slightly more in xylose than in glucose. On the other hand, growth rate inhibition by succinic and ferulic (Figure 3B), itaconic, oxalic, ferulic acid, and the inorganic acids (ammonium chloride, sulfate, phosphate or nitric acid), HMF, furfural, 4-hydroxybenzaldehyde or ethanol, was not sugar specific (Table 1). Clearly, the growth inhibition for some of the inhibitors is substrate specific and significant inhibitions were observed using xylose as the carbon source. At high hydrolysate concentrations, ethanol yields on xylose are lower than those on glucose for *Z. mobilis*[21]; these results may lead to new insights for strain improvement on xylose utilization.

Relative toxicities for xylose and glucose were calculated by dividing the mean inhibitor millimolar concentrations in glucose (IC_25_, IC_50_, IC_75_, and IC_100_) by the comparable values in xylose. These are presented in Table 2 along with pK_a_ values. Interestingly, the organic acids, which affected growth in xylose the most, were monocarboxylic acids. Dicarboxylic acids had a lesser effect on growth differences between xylose and glucose.

**Table 2:** **pK**_**a **_**values of compounds and relative toxicities for glucose and xylose were calculated by dividing the mean of inhibitor concentrations at IC**_**25**_**, IC**_**50**_**, IC**_**75 **_**and IC**_**100 **_**when grown in glucose divided by the mean inhibitor concentration at respective IC when grown in xylose**

**Compound**	**pK**_**a**_	**Average ratio of IC in RMG/IC in RMX**
**Organic acids**		
Levulinc acid	4.59	4.5 +/- 0.5
Benzoic acid	4.18	4.1 +/- 1.3
Acetic acid	4.76	3.7 +/- 0.4
Formic acid	3.75	3.2 +/- 0.8
Caproic (Hexanoic) acid	4.85	2.2 +/- 0.4
Lactic acid	3.86	1.9 +/- 0.4
2-Furoic acid	3.16	1.9 +/- 0.4
Vanillic acid	4.31, 8.81	1.4 +/- 0.1
Ferulic acid	4.56, 8.65	1.4 +/- 0.1
4-Hydroxycinnamic acid	4.63, 9.58	1.3 +/- 0.2
4-Hydroxybenzoic acid	4.48, 9.32	1.1 +/- 0.06
Itaconic acid	3.85, 5.45	0.9 +/- 0.1
Succinic acid	4.16, 5.61	1.0 +/- 0.2
Oxalic acid	1.27, 4.27	0.9 +/- 0.04
**Inorganic acids**		
Hydrochloric acid		0.8 +/- 0.2
Sulfuric acid		0.7 +/- 0.1
Phosphoric acid		0.6 +/- 0.1
Nitric acid		1.1 +/- 0.1
**Aldehydes**		
5-Hydroxymethylfurfural (HMF)	-0.37	1.0 +/- 0.1
Furfural	0.41	0.8 +/- 0.1
Syringaldehyde	0.99	0.9 +/- 0.1
Vanillin	1.21	1.5 +/- 0.4
4-Hydroxybenzaldehyde	1.35	1.6 +/- 0.1
**Alcohols**		
Furfuryl alcohol	0.28	1.0 +/- 0.1
Ethanol	-0.24	1.0 +/- 0.1

The mechanism of toxicity for weak acids for bacteria and yeast is shown to be due to the passage of the protonated form of the acid, which is highly permeable, through the cell membrane [33-35]. Cells lose energy if the acid is deprotonated in the higher pH of the cytoplasm (loss of protonmotive force) or if the acid is actively pumped out of the cell. The intracellular pH for *Z. mobilis* has been reported to be between 5.4- 5.6 in pH 5 medium in *Z. mobilis* 113 [36] and 6.2-5.3 for *Z. mobilis* CP4 during batch fermentation [37]. This is above the pK_a_ for the weak monocarboxylic acids evaluated in this study thus allowing for the disassociation of acid within the cell, resulted in a subsequent drop in internal pH and disruption of the transmembrane potential. Dicarboxylic acids with pK_a_ values above and below physiological pH are expected to be partially protonated and therefore less toxic due to lower permeability through the cell membrane. The observation that monocarboxylic acids were more inhibitory in xylose-grown cells than in glucose-grown cells was unexpected. The increased inhibitory action of monocarboxylic acids on xylose growth may possibly be the result of an already reduced energy state from inefficient xylose metabolism compounded by further disruption of the transmembrane potential, although further investigation is needed to support this hypothesis.

### Cation effect on inhibitor toxicity

We also examined the inhibitory profiles for four inorganic acids and two organic acids (sulfate, chloride, phosphate, nitrate, acetate and formate) using different cations, in order to determine what contribution cations have in hydrolysate toxicity. Both ammonia and calcium have been used in neutralization and conditioning steps following pretreatment and prior to saccharification and were examined along with potassium and sodium. Calcium sulfate and phosphate were not tested due to their low solubilities.

**Figure 4:**
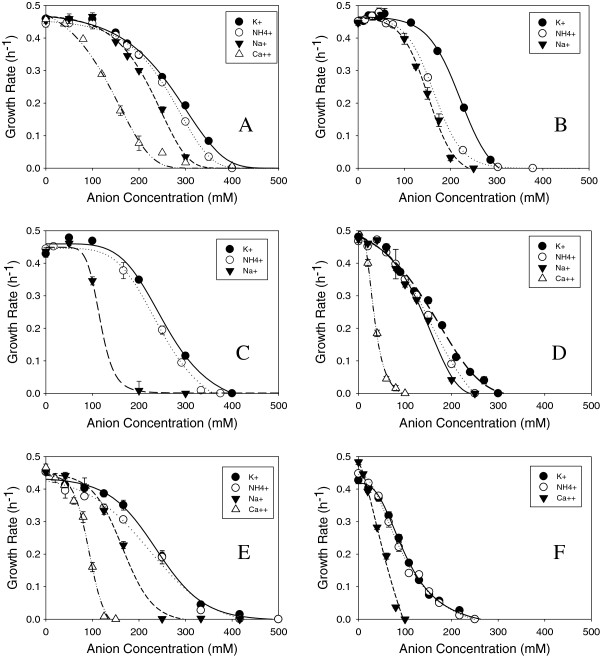
**Growth inhibition profiles of *Z. mobilis *8b using glucose as the substrate with different anions A) chloride, B) sulfate, C) phosphate, D) nitrate, E) acetate and F) formate neutralized to pH 5.8 using different cations.**

Figure 4A- F depicts growth inhibition profiles of *Z. mobilis* using glucose as the substrate in different salts of chloride, sulfate, phosphate, nitrate, acetate and formate, respectively. In most cases, potassium salts were the least inhibitory, followed by ammonium, sodium and calcium salts. Calcium is divalent and would be present at half the concentration of monovalent cations; yet it is quite inhibitory, particularly when paired with chloride, nitrate and formate.

**Figure 5:**
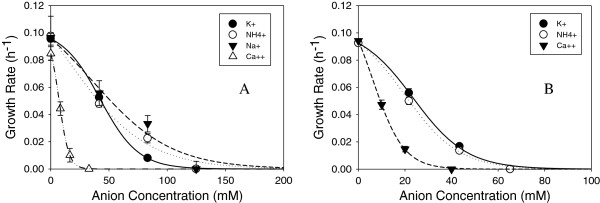
**Growth rate inhibition profiles of *Z. mobilis *8b using xylose as the substrate with different anions A) acetate and B) formate neutralized to pH 5.8 using different cations.**

The pattern of cation effect with xylose as the substrate follows a similar toxicity ranking where Ca^++^ > > Na^+^ > NH4^+^ > K^+^. Inhibitory profiles for acetate and formate in xylose cultures are shown in Figure 5A and B. Both acetate and formate cause reduced relative growth rates in xylose. Whereas the MIC for calcium acetate in glucose medium is 150 mM (300 mM acetate), the MIC in xylose medium is only 60 mM (120 mM acetate). Likewise, the MIC for calcium formate is 100 mM in glucose and only ~ 40 mM in xylose.

The examination of inorganic acids arose from the need to understand the contribution of salts towards hydrolysate toxicity that are introduced during pretreatment and subsequent neutralization/conditioning steps. Approximately, 250 to 400 mM of the anions (chloride, sulfate, nitrate and phosphate) caused complete inhibition of growth. An analysis of some dilute acid corn stover pretreated material conditioned with ammonium hydroxide used in a previous report [22] revealed concentrations of sulfate up to 200 mM would cause approximately 75% growth inhibition by itself without additional inhibitors. Lower acid pretreatment conditions reduce the introduction of inorganic salts and can benefit ethanologen growth and fermentation (unpublished results) though the lower severity pretreatment can have a negative impact on cellulose and hemicellulose hydrolysis.

In our analysis of cations, we discovered calcium to be the most toxic, followed by sodium, ammonium and potassium. Calcium overliming has been shown to provide advantages at least in part through the reduction in the levels of sulfate, HMF and furfural as well as other phenolics, but at the same time introduces new inhibitors, especially calcium ions that are particularly inhibitory in *Z. mobilis*, when paired with acetate or formate. Calcium’s role in toxicity may arise from its ability to form a wide variety of complexes in biological systems and compete directly with magnesium for many important enzymes [27-30]. Calcium toxicity may explain why *Z. mobilis* performs better in ammonium conditioned hydrolysates rather than in calcium overlimed hydrolysates [38].

### Effect of combined acetate, formate, HMF and furfural on *Z. mobilis* growth in glucose and xylose

We investigated inhibitory synergism among four compounds (HMF, furfural, acetate and formate), chosen because of their inhibitory potential and concentration in acid pretreated corn stover hydrolysate. We performed a two-level, four-factor factorial design using growth rate as the response factor and used concentrations of inhibitors which would cause 20% inhibition for each based on glucose substrate (10 mM HMF, 5 mM furfural, 125 mM acetate and 50 mM formate). Two center points were included containing half the concentrations above combining all the inhibitors.

**Figure 6:**
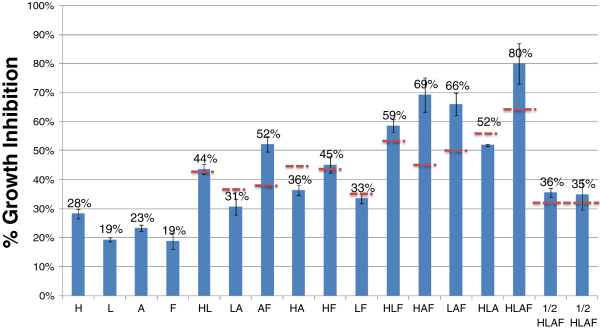
**Growth inhibitions as a percentage of growth rate without inhibitor subtracted from 100% at concentrations of each inhibitor causing approximately 20% inhibition H = 10 mM HMF, L = 5 mM furfural, A = 125 mM acetate, F = 50 mM formate.** The dashed lines represent the inhibitory level if inhibitions were additive. 1) H; 2) L; 3) A; 4) F; 5) HL; 6) LA, 7) AF; 8) HA; 9) HF; 10) LF; 11) HLF; 12) HAF; 13) LAF; 14) HLA; 15) HLAF; 16-19) ½ of the levels of all compounds (HLAF).

The results are shown in Figure 6 as the percent of inhibition from the control without the inhibitor. The first four bars represent the percent inhibition caused by each of the four compounds individually: HMF (H), furfural (L), acetate (A) and formate (F), respectively. The presence of each compound caused ~ 20% growth inhibition, compared to the control, except for HMF which caused 28% inhibition of growth. The dashed line indicates theoretical levels of inhibition should the effect of the toxins be additive. For example, since the growth rate in HMF is 72% that of the control and in furfural, 81% of the control, the combination would result in 72% × 81% = 58% of the control growth rate, or 42% growth rate inhibition. Experimentally, we obtained 44% inhibition for the combination of HMF and furfural, indicating that both inhibitors did not act synergistically. In the presence of acetate and formate, we did see significant deviations from expected inhibitions, obtaining from 16-24% higher growth rate inhibitions with combinations of AF, HAF, LAF and HLAF. Results were also analyzed by Design Expert Version 7 from Stat-Ease, Inc. (Minneapolis, MN), which indicated that an interaction between acetate and formate is significant. (Additional file 4: Figure S4), however even though the model was significant, lack of fit was also noteworthy, indicating that more data points are needed to confirm this hypothesis.

Combinations of HMF, furfural and acetate resulted in additive rather than synergistic inhibition. These results differ from published results using *E. coli* in which furfural and HMF were shown to act synergistically with a number of compounds [4,17]. In *S. cerevisiae* strain 259ST, however, furfural, ethanol and acetate combinations were found to have additive inhibition to growth but synergistic inhibition for ethanol production [24]. Further investigation is needed to determine whether ethanol production is synergistically inhibited in *Z. mobilis* by these compounds and whether some of the other compounds act synergistically to inhibit growth and/or ethanol production.

### Aldehyde conversion by *Z. mobilis*

**Figure 7:**
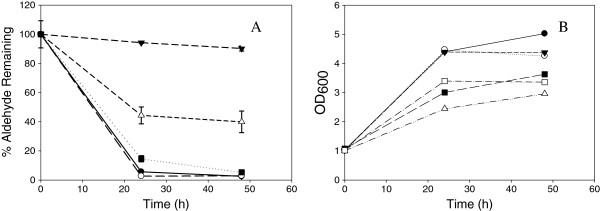
**A) Aldehyde disappearance in shake flask fermentations of *Z. mobilis *8b containing inhibitor at IC**_**50 **_**and B) corresponding growth curves monitoring absorbance for the following inhibitors: HMF (●),furfural (○),syringaldehyde (▼),vanillin (**Δ**) and 4-hydroxybenzaldehyde (■).**

We observed the reduction of HMF and furfural by *Z. mobilis* during earlier fermentation studies conducted in our laboratory which has been previously reported [13]. High cell density inocula could improve fermentation performance by reducing the impact of toxic compounds, particularly if compounds were converted to a less toxic compound or intermediates. We evaluated compound conversions in shake flasks, using a high cell inoculum at concentrations of aldehydes, which cause 50% of growth inhibition. The concentrations of HMF, furfural, syringaldehyde, vanillin and 4-hydroxybenzaldehyde used for this experiment were 21.4, 15.6, 17.6, 4.3, and 5.7 mM, respectively. After 24 hours, all the sugar was consumed and most cultures reached stationary phase (Figure 7B) at final cell densities lower than the control (Figure 7B). After 48 hours, 97% of HMF or furfural had disappeared (Figure 7A). Surprisingly, *Zymomonas* also has the ability to metabolize most of 4-hydroxybenzaldehyde (95%) and 60% of vanillin after 48 hours. In control flasks without cells, most of the compounds remained at initial levels after 48 hours, except for vanillin and syringaldehyde which showed a loss of 14-16% over 48 hours, respectively. The amount of syringaldehyde removed with cells was at 10%, less than the abiotic control. The near-complete elimination of HMF, furfural, and 4-hydroxybenzaldehyde and partial elimination of vanillin occurred simultaneously with the appearance of single unique peaks on UV chromatograms (Additional file 1: Figure S1). The product of furfural conversion eluted at the same retention time and had the same spectral profile in the UV range (between 230 nm – 340 nm) as furfuryl alcohol. Likewise, the apparent conversion products of vanillin and 4-hydroxybenzaldehyde eluted at the same retention times and had the same spectral profile as vanillyl and 4-hydroxybenzyl alcohol. Confirmatory evidence that the conversion products were the alcohol analog of the added aldehydes was provided by GC-MS analysis. (Additional file 2: Figure S2). The conversion of the aldehyde to its alcohol form implies a reductive mechanism for conversion. Since the alcohol form of HMF is not commercially available, nor the mass spectrum available for this compound, we could not conclude that a reductive conversion pathway was operating for this compound, though it seems quite likely.

### Fermentation performance by *Z. mobilis* 8b in glucose and xylose in the presence of inhibitors at 1X MIC

Complete inhibition of growth does not necessarily reflect cell death nor inhibition of fermentation, particularly with more concentrated inocula. To investigate whether ethanol could still be produced at inhibitor concentrations that prevent growth, we conducted small scale fermentation experiments (4 mL) in the presence of either 5% glucose or 5% xylose over a 24 or 48 hour period for glucose and xylose, respectively.

**Figure 8:**
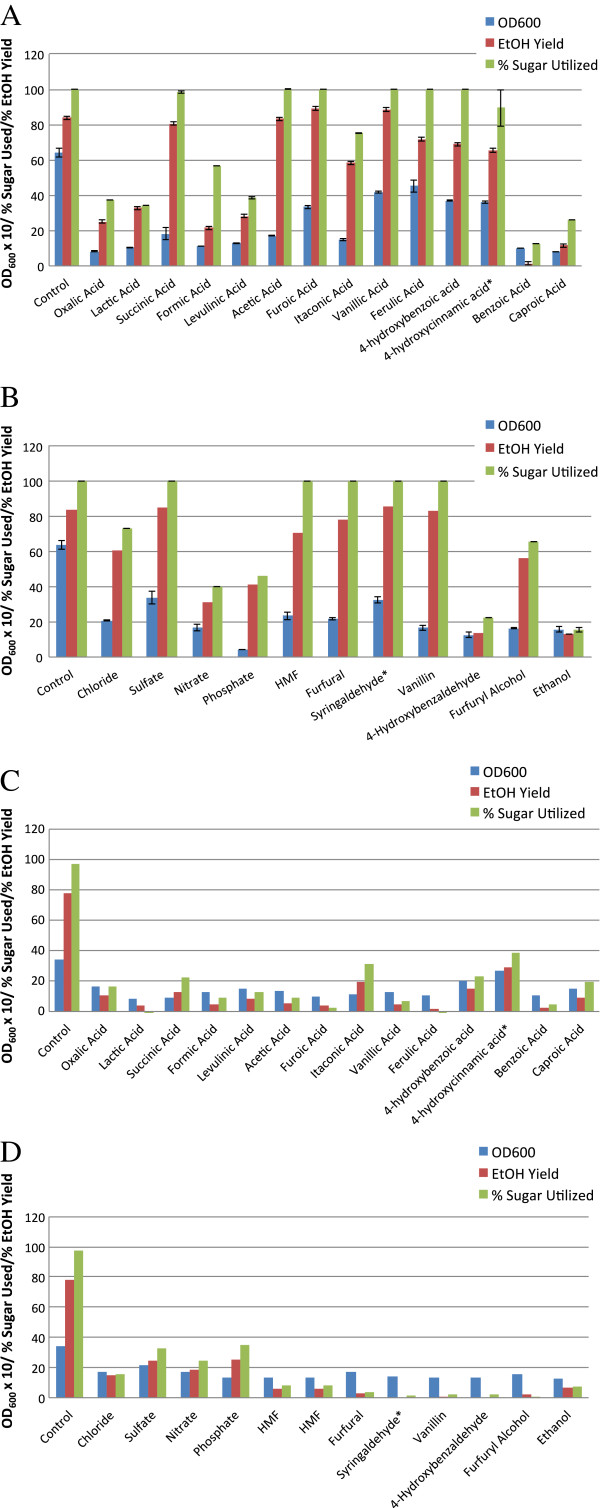
**Mini-fermentation analysis of Z. *mobilis *8b in the presence of model inhibitor compounds at 1X MIC and glucose (A and B) or xylose (C and D), plotting OD**_**600**_**, ethanol yields and percent of sugar utilization.**

Data are presented in Figure 8 for *Z. mobilis* 8b grown in the presence of organic acids, aldehydes, inorganic salts and alcohol at 1X MIC in both glucose (A and B) and xylose (C and D). We observed a wide range of growth and fermentation responses for *Z. mobilis* in inhibitor cultures grown in glucose. In the following compounds, there was little or no growth (OD_600_ < 2.0) or fermentation (theoretical ethanol yield < 50%) observed: oxalic, benzoic, caproic, lactic, levulinic, formic, itaconic, phosphoric, and nitric acids; 4-hydroxybenzaldehyde, furfuryl alcohol and ethanol. The following completely inhibited growth but did not completely block ethanol production: acetic, succinic and hydrochloric acids. For the remainder of the inhibitors tested for glucose fermentations, we observed both growth and fermentation at IC_100_: ferulic, furoic, vanillic, 4-hydroxybenzoic, 4- hydroxycinnamic, and sulfuric acids in addition to syringaldehyde, vanillin, HMF and furfural.

When xylose is used in place of glucose as the substrate, growth rates and final cell mass are significantly reduced [21,22,39]. Even with no inhibitors present, *Z. mobilis* 8b was only able to reach a final cell density of ~ 2.7, less than one half of the cell density achieved from glucose cultures. Approximately 97% of the available xylose was consumed and produced 83% of theoretical ethanol yields. Ethanol yields were lower than typically observed in pH controlled fermentations which could be attributed to the lack of pH control. Although some inhibitors caused uncoupled ethanol production from growth in glucose, all compounds that completely inhibited growth in xylose also inhibited ethanol production. Some growth was evident in the presence of 4-hydroxybenzoic and 4-hydroxycinnamic acid from 48 to 72 hours following an initial lag with concomitant ethanol production. None of the other cultures exhibited a similar lag of growth or fermentation.

In mini-fermentations, *Z. mobilis* 8b was able to grow and produce ethanol at 1X MIC in the remaining compounds (ferulic, furoic, vanillic, 4-hydroxybenzoic, 4- hydroxycinnamic, and sulfuric acids in addition to syringaldehyde, vanillin, HMF and furfural), although final cell densities were lower than observed with the control. One possible explanation might be that the presence of high cell concentration might enhance the capacity to bind extracellular inhibitors, thus reducing the bioavailability. It is also possible that the intracellular concentration of these inhibitors is lower when they are distributed among a larger number of cells. It is also conceivable that growth conditions are different enough in ini-fermentations compared to Bioscreen C growth assays to account for differences. As described earlier, *Z. mobilis* is capable of converting some inhibitors to a less toxic product. This appears to be the case with the apparent reduction of key aldehydes to the corresponding alcohol. This phenomenon was also observed in fermentation experiments conducted in the presence of aldehydes added at the IC_50_ concentration. Z*. mobilis* was capable of reducing toxic amounts furfural, vanillin, 4-hydroxybenzaldehyde and some syringaldehyde to the apparent alcohol product. Although *Z. mobilis* 8b was able to convert 4-hydroxybenzaldehyde, it was not able to overcome growth and fermentation inhibition at 1X MIC. Further investigation will be needed to determine whether *Z. mobilis* is capable of converting the other phenolics studied in this report. Previous reports have observed the reduction of HMF and furfural concentrations during fermentation in shake flask studies with *Z. mobilis* ATCC 10988 [13]. We will be conducting additional studies to determine the gene(s) responsible for conversion of these aldehydes. Enhanced conversion of toxic aldehydes with engineered strains could reduce process costs by improving ethanol yields or by reducing fermentation time.

### Comparison of *Zymomonas* to other ethanologens

Although some preliminary studies have evaluated the effects of model compounds present in hydrolysates on *Z. mobilis*, the strains, media and methods used to assess toxicity varied significantly [11-14]. The data are compiled and presented in Additional file 5: Table S1 for comparison purposes. Since the ranges of concentrations were limited in previous publications, information on MIC has not been reported. From our examination, *Z. mobilis* 8b performed better in acids and aldehydes using growth as the criteria than when compared with *E. coli*, *Thermoanerobacter mathranii*, *Candida shehateae*, and *Pichia stipitis*[7,16,17].

*Z. mobilis* 8b, derived from *Z. mobilis* ZM4, performed much better than *Z. mobilis* CP4/pZB5 [11-14] and ZM4 (ATCC31821) has been shown to perform much better in ethanol fermentations than CP4 [40,41]. Thus it is not surprising that 8b outperformed better in HMF, furfural, vanillin, syringaldehyde and acetic acid. Similar tolerance levels to HMF, furfural, vanillin and acetate were observed for *Z. mobilis* 10988, ZM4 and 8b [11,13,14,21,42].

What was a surprising observation in this study and has not been previously demonstrated, was that sensitivities to monovalent acids, was particularly strong when xylose was used as the sole substrate. Thus, the challenges of engineering a strain for efficient utilization of pentose sugars are compounded by the additional burden of increased toxicity by these common inhibitors. Clearly, future genetic engineering efforts of *Z. mobilis* should be directed towards improving growth and thus fermentation performance in organic acid (particularly acetate) through an understanding of the mechanism of organic acid toxicity in both glucose and xylose.

## Conclusions

This is the first comprehensive report on the inhibitory effects of model compounds on *Z. mobilis* using a systematic approach based on inhibitory growth profiles. Using data obtained in this report, we have established a toxicity database for various compounds. Combined with analysis of hydrolysate composition, we are able to make predictions on the contribution of each compound towards toxicity and can begin to prioritize inhibitors based on their relative importance to overall toxicity in hydrolysates for microorganisms. Guidance can then be provided, accordingly, for potential process development, along with potential future strain improvement and tolerance strategies.

## Methods

### Reagents and strains

The *Z. mobilis* strain used in this study was the recombinant strain 8b engineered for xylose utilization [20]. The following chemicals were obtained from Sigma-Aldrich (St. Louis, MO): calcium acetate, calcium nitrate, succinic acid, sodium sulfate, sodium chloride, ammonium chloride, potassium chloride, calcium chloride, 2-furoic acid, ferulic acid, formic acid, L (+) lactic acid, potassium nitrate, ammonium nitrate, monobasic ammonium phosphate, dibasic potassium phosphate, furfural, syringaldehyde, MES (4-morpholineethanesulfonic acid) and methyl sulfoxide (DMSO), levulinic acid, benzoic acid, potassium sulfate, hexanoic (caproic) acid, 4-hydroxycinnamic (ρ-coumaric) acid, 4-hydroxybenzoic acid, vanillic acid, oxalic acid, furfuryl alcohol, HMF (5-hydroxymethylfufural), vanillin and 4-hydroxybenzaldehyde. The following chemicals were obtained from JT Baker: acetic acid, ammonium sulfate, dibasic ammonium phosphate, and monobasic potassium phosphate.

### Culture conditions and growth rate studies

Cultures were grown in RM medium (10 g/L yeast extract, 2 g/L KH_2_PO_4_) supplemented with either 2% (w/v) glucose (RMG) or 2% (w/v) xylose (RMX) for inhibitor studies. The pH of all media was adjusted to pH5.8 and was filter sterilized. Media were prepared from stock solutions of yeast extract and monobasic potassium phosphate, and when possible, inhibitor stock solutions were prepared and titrated to pH 5.8. Two compounds (4-hydroxycinnamic acid and syringaldehyde) were prepared from stock solutions in 100% DMSO due to their low solubility in water. The total amount of DMSO in the final medium ranged from 0.1-5.0% (v/v) for 4-hydroxycinnamic acid and 0.1%-3% (v/v) in syringaldehyde. Inhibitor studies with DMSO alone did not detect notable inhibitions on growth or final cell mass (data not shown).

Ammonium salts were prepared by titrating the acids with concentrated ammonium hydroxide, except for the anions: sulfate, chloride, nitrate, acetate, phosphate. Calcium formate was prepared by titrating formic acid with lime. Phosphate stocks were made by preparing 1 M stock solutions of monobasic phosphate and titrating the medium to pH of 5.8 with 1 M dibasic solution.

As a consequence of precipitation of monobasic potassium phosphate with calcium, it was not included in medium containing calcium. In this case, 50 mM MES, pH 5.8, was provided to supply some buffering capacity. Growth rates in RMG or RMX with 50 mM MES, pH 5.8, and no potassium phosphate were similar to those obtained in media with potassium phosphate which indicated that yeast extract in rich media could supply sufficient phosphorous for growth at the 2% sugar level (data not shown).

Overnight cultures in RMG medium were either started from single colonies or from glycerol stocks. Optical densities were measured using a Beckman DU-640 spectrophotometer (Beckman Coulter, Inc., Brea, CA) for inoculation. Growth rates were obtained from the Bioscreen C analyzer purchased from Growth Curves USA (Piscataway, NJ). Procedures for inoculation, growth conditions, measurement, recording of final cell densities and calculations used to correct for non-linear response at high cell densities were previously reported [22]. In brief, log phase cultures of *Z. mobilis* 8b (a recombinant xylose-utilizing strain of ZM4) were obtained by inoculating overnight cultures in RMG at 30°C and allowing the cells to grow to an OD_600_ ~ 1.0. Cells were then spun down at 3840 × g, for 10 min at RT and resuspended in RMG or RMX with inhibitor at the desired concentration such that the starting cell density distributed to Bioscreen C microplates after appropriate dilutions with inhibitors was OD_600_ = 0.05 (~5 × 10^6^ cells/mL^=^) in a total volume of 300 μL. Incubations were performed at 30°C and absorbance readings were taken every 10 min. Operation of the Bioscreen C and collection of turbidity measurements (OD_420–580_) were computer automated with EZ Experiment. Data were collected and exported to Microsoft Excel spreadsheets.

Cultures for mini-fermentation studies at 1X MIC were inoculated with *Z. mobilis* 8b from seed cultures at an OD_600_ of 1.0 described above, in 4.5 mL of RM medium containing 5% glucose or 5% xylose and inhibitor compounds at a concentration which would cause 100% inhibition of growth rates (1X MIC) in 6 mL HPLC vials at 30°C, 150 rpm, and were vented with an 18 gauge needle and 0.2 micron syringe filter. Samples (0.5 mL) were removed at 0, 24 and 48 hours post inoculation for OD_600_ and HPLC analysis.

Cultures for aldehyde conversion studies were inoculated with *Z. mobilis* 8b at an OD_600_ of 1.0 in 100 mL of RMG containing 5% glucose in 125 mL unbaffled shake flasks containing aldehyde inhibitors at a concentration that would cause 50% inhibition of growth rates at 30°C, 125 rpm. Samples were removed at 0, 24 and 48 hours for HPLC and growth analysis. Flasks containing inhibitor medium without cells were included to assess abiotic loss due to instability or volatility.

### High performance liquid chromatography (HPLC) analysis of aldehydes and aldehyde conversion products

Aldehydes and their conversion products were analyzed by reverse-phase HPLC with photodiode array detection (Agilent 1100, Agilent Technologies, Santa Clara, CA) on a C-18 column (Phenomenex, Luna, 150 × 4.6 mm; Torrance, CA) using a linear gradient of acetonitrile-water over 35 min at a flow rate of 0.5 mL/min.

### Gas chromatography-mass spectroscopy (GC/MS) analysis of aldehydes and aldehyde conversion products

Aldehydes and their conversion products from same samples analyzed above were also analyzed by GC/MS on an Agilent 6890 GC equipped with a 5973 MS (Agilent Technologies, Palo Alto, CA). Sample compounds were separated using a HP-5MS column (Agilent 122-5532, 30 m × .25 mm × .25 μm HP-5MS column). HP MSD Chemstation software (Agilent) equipped with NIST database Rev. D.03.00 was used to determine the identity of the unknown compounds found within the samples. Each sample was placed on an auto-sampler (Agilent) and injected at a volume of 1 uL. The GC/MS method consisted of a front inlet temperature of 285°C, MS transfer line temperature of 280°C, and a scan range from 35 m/z to 450 m/z. A constant flow of 1 ml/min was held throughout the run. A starting temperature of 35°C was held for 3 minutes and then ramped at 15°C/min to a temperature of 225°C and held for 1 minute, then continued at a ramped rate of 15°C/min to 300°C and held for 5 minutes. The method resulted in a run time of 26.67 minutes for each sample.

## Abbreviations

HMF: 5-hydroxymethylfurfural; Ca: Calcium; Na: Sodium; NH4: Ammonium; K: Potassium; MIC: Minimum inhibitor concentration; M: Molar; mM: Millimolar; g/L: Gram per liter; L: Liter; mL: Milliliter; min: Minute; ICx: Inhibitor concentration causing x% growth inhibition; MES: 4-morpholineethanesulfonic acid; DMSO: Methyl sulfoxide; RM: Rich medium; HPLC: High performance liquid chromatography; OD600: Optical density at 600 nanometers.

## Competing interests

The authors declare that they have no competing interests.

## Authors’ contributions

MZ conceived the ideas and directed the overall project. MZ and MAF designed experiments and MAF conducted and analyzed all experimental work. AM performed mini-fermentation assays. HP performed HPLC analysis of aldehydes. PP provided technical oversight and reviewed the manuscript. All authors have read and approved the final manuscript.

## Supplementary Material

Additional file 1: Figure S1 HPLC chromatograms of samples taken with *Z. mobilis* 8b cells at t = 0 hrs (blue) with aldehydes and overlaid with samples taken at t = 24 hrs (red) after conversion of aldehydes and overlaid with a standard of the pure alcohol compound: **A)** with furfural at t0 (260 nm), t24 (210 nm) and 0.1 g/L furfuryl alcohol (210 nm); **B)**, with syringaldehyde at t0 (260 nm), t24 (210 nm) and 1 g/L syringyl alcohol (210 nm); C), with vanillin at t0 (210 nm), t24 (210 nm) and 1 g/L vanillyl alcohol (260 nm); D) and with 4-hydroxybenzaldehyde at t0 (210 nm), t24 (210 nm) and 1 g/L 4-hydroxybenzyl alcohol (260 nm). The spectrum of peak co-eluting with alcohol compound was overlaid with sample at t24 hrs and embedded in corresponding chromatogram figures. Click here for file

Additional file 2: Figure S2 GC chromatograms of samples taken with *Z. mobilis* 8b cells at t = 0 hrs (black) with aldehydes and at t = 24 hrs (blue) after conversion of aldehydes: cell control with no aldehyde compound **(A)**, 5-HMF **(B)**, furfural **(C)**, syringaldehyde **(D)**, vanillin **(E)** and 4-hydroxybenzaldehyde **(F)**. MS confirmed the identity of the compound and its conversion product with > 90% confidence. Click here for file

Additional file 3: Figure S3 Growth rates (●) and relative final cell densities (∆) of *Z. mobilis* 8b grown in glucose and **A)** ammonium formate, **B)** ammonium itaconate **C)** ammonium 4-Hydroxybenzoate and **D)** ammonium sulfate. Click here for file

Additional file 4: Figure S4 Interaction model for acetate and formate on the growth rate of *Z. mobilis* 8b in glucose. Click here for file

Additional file 5: Table S1 Inhibition of growth and ethanol yield in *Z. mobilis* obtained from the literature. Click here for file
