# Supplementary material for: Inhibition of growth of Zymomonas mobilis by model compounds found in lignocellulosic hydrolysates
Source: Biotechnol Biofuels. 2013 Jul 9;6:99. doi: 10.1186/1754-6834-6-99 (PMC3716709; doi:10.1186/1754-6834-6-99)
Supplement: Additional file 1: Figure S1 — HPLC chromatograms of samples taken with Z. mobilis 8b cells at t = 0 hrs (blue) with aldehydes and overlaid with samples taken at t = 24 hrs (red) after conversion of aldehydes and overlaid with a standard of the pure alcohol compound: A) with furfural at t0 (260 nm), t24 (210 nm) and 0.1 g/L furfuryl alcohol (210 nm); B), with syringaldehyde at t0 (260 nm), t24 (210 nm) and 1 g/L syringyl alcohol (210 nm); C), with vanillin at t0 (210 nm), t24 (210 nm) and 1 g/L vanillyl alcohol (260 nm); D) and with 4-hydroxybenzaldehyde at t0 (210 nm), t24 (210 nm) and 1 g/L 4-hydroxybenzyl alcohol (260 nm). The spectrum of peak co-eluting with alcohol compound was overlaid with sample at t24 hrs and embedded in corresponding chromatogram figures. [file 1754-6834-6-99-S1.docx]

**A**

**B**

**C**

**D**

**Additional file 1: Figure S1.** HPLC chromatograms of samples taken with *Z. mobilis* 8b cells at t=0 hrs (blue) with aldehydes and overlaid with samples taken at t=24 hrs (red) after conversion of aldehydes and overlaid with a standard of the pure alcohol compound: A) with furfural at t0 (260nm), t24 (210 nm) and 0.1 g/L furfuryl alcohol (210nm); B), with syringaldehyde at t0 (260 nm), t24 (210nm) and 1 g/L syringyl alcohol (210nm); C), with vanillin at t0 (210nm), t24 (210nm) and 1 g/L vanillyl alcohol (260nm); D) and with 4-hydroxybenzaldehyde at t0 (210nm), t24 (210nm) and 1 g/L 4-hydroxybenzyl alcohol (260nm). The spectrum of peak co-eluting with alcohol compound was overlaid with sample at t24 hrs and embedded in corresponding chromatogram figures.
