# Supplementary material for: Inhibition of growth of Zymomonas mobilis by model compounds found in lignocellulosic hydrolysates
Source: Biotechnol Biofuels. 2013 Jul 9;6:99. doi: 10.1186/1754-6834-6-99 (PMC3716709; doi:10.1186/1754-6834-6-99)
Supplement: Additional file 2: Figure S2 — GC chromatograms of samples taken with Z. mobilis 8b cells at t = 0 hrs (black) with aldehydes and at t = 24 hrs (blue) after conversion of aldehydes: cell control with no aldehyde compound (A), 5-HMF (B), furfural (C), syringaldehyde (D), vanillin (E) and 4-hydroxybenzaldehyde (F). MS confirmed the identity of the compound and its conversion product with > 90% confidence. [file 1754-6834-6-99-S2.docx]

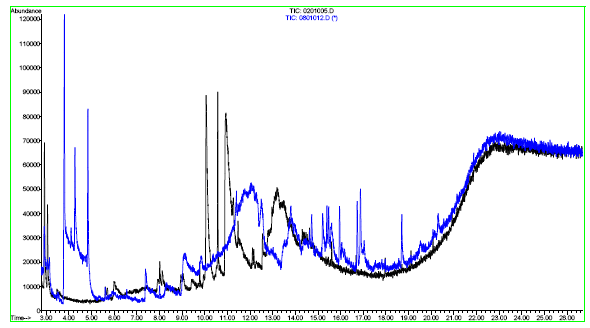


**A**


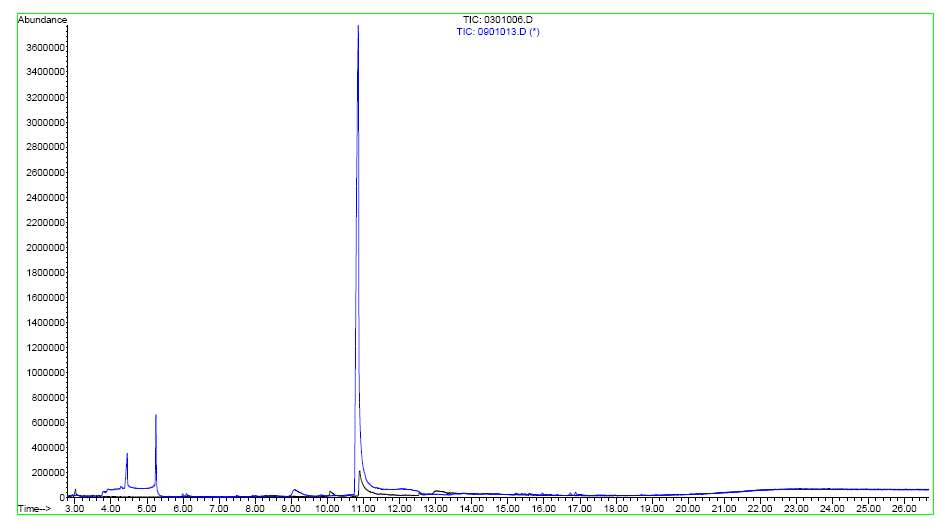


**B**

??

HMF


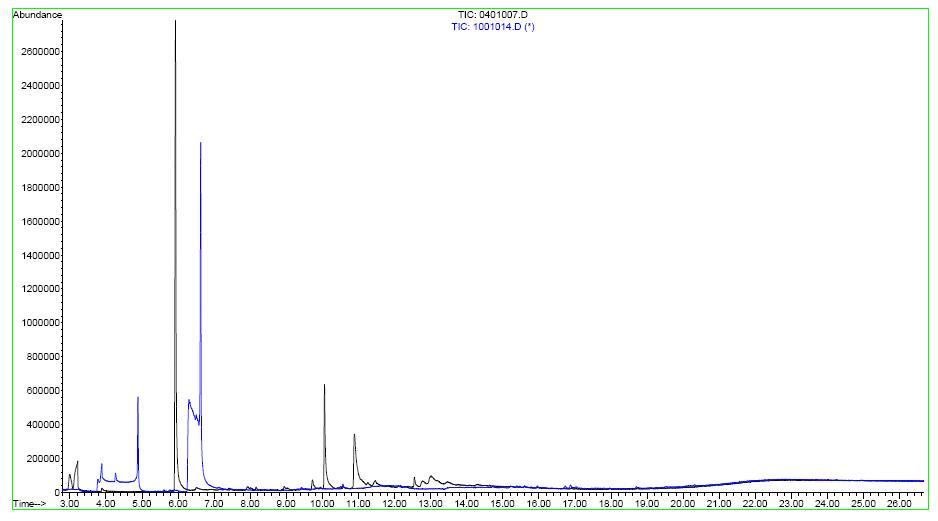


Furfural

Furfuryl alcohol

**C**


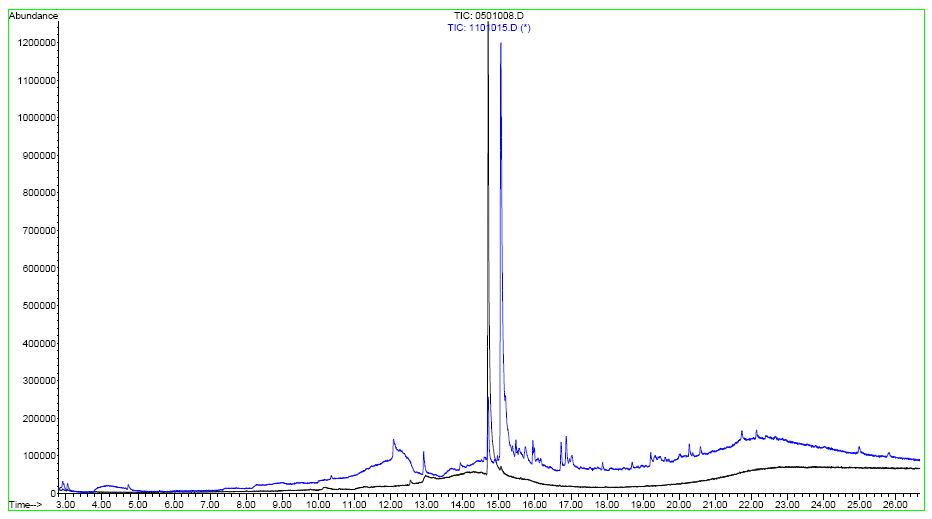


**D**

Syringyl alcohol

Syringaldehyde


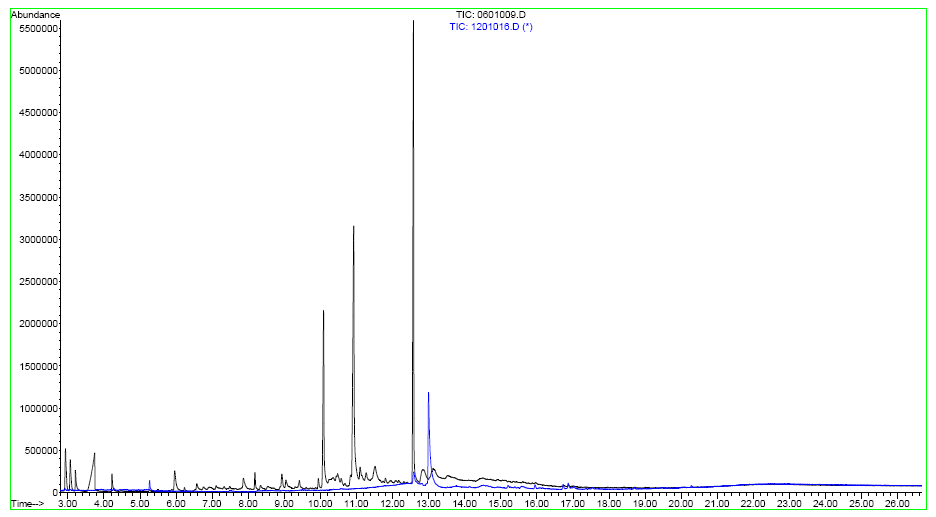


**E**

Vanillin

Vanillyl alcohol


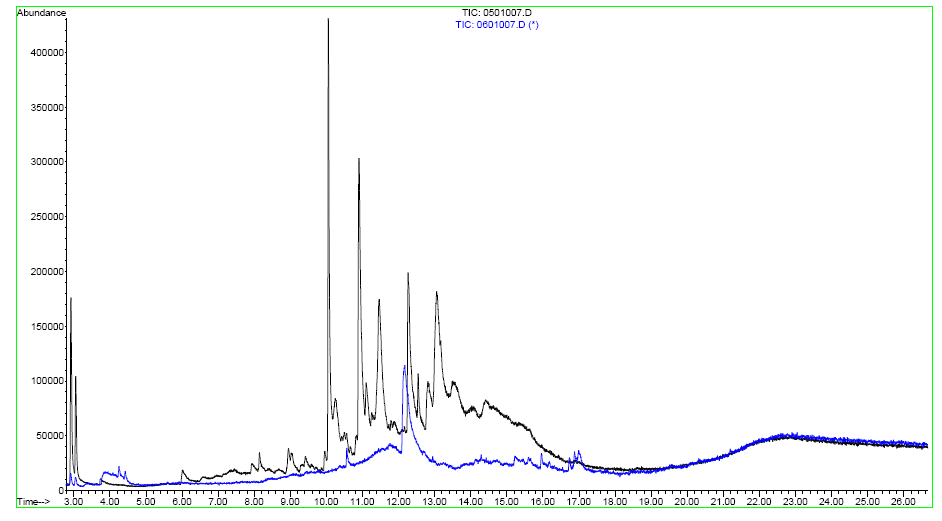


**F**

4-hydroxybenaldehyde

4-hydroxybenzyl alcohol

**Additional file 2: Figure S2**. GC chromatograms of samples taken with *Z. mobilis* 8b cells at t=0 hrs (black) with aldehydes and at t=24 hrs (blue) after conversion of aldehydes: cell control with no aldehyde compound (A), 5-HMF (B), furfural (C), syringaldehyde (D), vanillin (E) and 4-hydroxybenzaldehyde (F). MS confirmed the identity of the compound and its conversion product with > 90% confidence.
