# Supplementary material for: Inhibition of growth of Zymomonas mobilis by model compounds found in lignocellulosic hydrolysates
Source: Biotechnol Biofuels. 2013 Jul 9;6:99. doi: 10.1186/1754-6834-6-99 (PMC3716709; doi:10.1186/1754-6834-6-99)
Supplement: Additional file 3: Figure S3 — Growth rates (●) and relative final cell densities (∆) of Z. mobilis 8b grown in glucose and A) ammonium formate, B) ammonium itaconate C) ammonium 4-Hydroxybenzoate and D) ammonium sulfate. [file 1754-6834-6-99-S3.docx]

A

B

C

D

**Additional file 3: Figure S3**. Growth rates (●) and relative final cell densities (△) of *Z. mobilis* 8b grown in glucose and A) ammonium formate, B) ammonium itaconate C) ammonium 4-Hydroxybenzoate and D) ammonium sulfate
