# Supplementary material for: Inhibition of growth of Zymomonas mobilis by model compounds found in lignocellulosic hydrolysates
Source: Biotechnol Biofuels. 2013 Jul 9;6:99. doi: 10.1186/1754-6834-6-99 (PMC3716709; doi:10.1186/1754-6834-6-99)
Supplement: Additional file 4: Figure S4 — Interaction model for acetate and formate on the growth rate of Z. mobilis 8b in glucose. [file 1754-6834-6-99-S4.docx]

**Additional file 4: Figure S4**. Interaction model for acetate and formate on the growth rate of *Z. mobilis* 8b in glucose.
