# Supplementary material for: Inhibition of growth of Zymomonas mobilis by model compounds found in lignocellulosic hydrolysates
Source: Biotechnol Biofuels. 2013 Jul 9;6:99. doi: 10.1186/1754-6834-6-99 (PMC3716709; doi:10.1186/1754-6834-6-99)
Supplement: Additional file 5: Table S1 — Inhibition of growth and ethanol yield in Z. mobilis obtained from the literature. [file 1754-6834-6-99-S5.docx]

**Additional file 5: Table S1**. Inhibition of growth and ethanol yield in *Z. mobilis* obtained from the literature.

| **Inhibitor** | **Concentration (mM)** | **Growth Rate**  **(%)*** | **Ethanol Yield (%)^*^** | **Microorganism** | **Reference***** |
| --- | --- | --- | --- | --- | --- |
| 5-HMF | 0.8 |  | 80 | *Z. mobilis* CP4/pZB5 | Ranatunga et al., 1997 |
|  | 7 |  | 100 | *Z. mobilis* ZM4/pZB5 | Kim et al., 2000 |
|  | 8 | 51 | 85 | *Z. mobilis* ATCC 10988 | Delgenes et al., 1996 |
|  | 24 | 69 | 87 | *Z. mobilis* ATCC 10988 | Delgenes et al., 1996 |
|  | 40 | 33 | 47 | *Z. mobilis* ATCC 10988 | Delgenes et al., 1996 |
|  | 22 | 50 |  | *Z. mobilis 8b* | This study |
| Furfural | 10 |  | 58 | *Z. mobilis* CP4/pZB5 | Ranatunga et al., 1997 |
|  | 3 |  | 95 | *Z. mobilis* ZM4/pZB5 | Kim et al., 2000 |
|  | 5 | 82 | 96 | *Z. mobilis* ATCC 10988 | Delgenes et al., 1996 |
|  | 10 | 81 | 82 | *Z. mobilis* ATCC 10988 | Delgenes et al., 1996 |
|  | 21 | 44 | 56 | *Z. mobilis* ATCC 10988 | Delgenes et al., 1996 |
|  | 17 | 50 |  | *Z. mobilis 8b* | This study |
| Vanillin | 0.3 |  | 65 | *Z. mobilis* CP4/pZB5 | Ranatunga et al., 1997 |
|  | 0.3 |  | 100 | *Z. mobilis* ZM4/pZB5 | Kim et al., 2000 |
|  | 3.3 | 45 |  | *Z. mobilis* ZM4 | Chen et al., 2012 |
|  | 6.6 | 33 |  | *Z. mobilis* ZM4 | Chen et al., 2012 |
|  | 4 | 50 |  | *Z. mobilis 8b* | This study |
| Syringaldehyde | 0.7 |  | 64 | *Z. mobilis* CP4/pZB5 | Ranatunga et al., 1997 |
|  | 0.7 |  | 100 | *Z. mobilis* ZM4/pZB5 | Kim et al., 2000 |
|  | 18 | 50 |  | *Z. mobilis 8b* | This study (in glucose) |
|  | 10 | 50 |  | *Z. mobilis 8b* | This study (in xylose) |
| Acetic Acid | 150 |  | 0 | *Z. mobilis* CP4/pZB5 | Ranatunga et al., 1997 |
|  | 181 |  | 100 | *Z. mobilis* ZM4/pZB5 | Kim et al., 2000 |
|  | 83 | 76 | 90 | *Z. mobilis* ATCC 10988 | Delgenes et al., 1996 |
|  | 167 | 44 | 102 | *Z. mobilis* ATCC 10988 | Delgenes et al., 1996 |
|  | 250 | 26 | 83 | *Z. mobilis* ATCC 10988 | Delgenes et al., 1996 |
|  | 181 |  | 100 | *Z. mobilis* ZM4/pZB5 | Kim et al., 2000 |
|  | 67 | 94 |  | *Z. mobilis* ZM4 | Joachimsthal et al., 1998 |
|  | 133 | 92 |  | *Z. mobilis* ZM4 | Joachimsthal et al., 1998 |
|  | 200 | 60 |  | *Z. mobilis* ZM4 | Joachimsthal et al., 1998 |
|  | 266 | 0 |  | *Z. mobilis* ZM4 | Joachimsthal et al., 1998 |
|  | 133 | 78 | 103 | *Z. mobilis 8b* | Mohagheghi et al., 2004 |
|  | 266 | 28 | 91 | *Z. mobilis 8b* | Mohagheghi et al., 2004 |
|  | 286 | 0 |  | *Z. mobilis ATCC 29191* | Fein et al., 1984 |
|  | 166 | 60 |  | *Z. mobilis* ZM4 | Chen et al., 2012 |
|  | 200 | 30 |  | *Z. mobilis* ZM4 | Chen et al., 2012 |
|  | 210 | 50 |  | *Z. mobilis 8b* | This study (in glucose) |
|  | 50 | 50 |  | *Z. mobilis 8b* | This study (in xylose) |
|  | 110 | 100 |  | *Z. mobilis 8b* | This study (in xylose) |
|  |  |  |  |  |  |

* Growth Rate: % of growth rate compared to control, except for ZM4 results (Joachimsthal et al., 1998). These were determined by comparing growth rates in acid to control growth rate.

** Ethanol Yield as a percent of control

*** Growth Conditions used in references:

Ranatunga et al., 1997: 10 mL shaken tube, 30^o^C, initial pH 6.0, 0.2 inoculum OD, 2.4 g/L glucose and 44.7 g/L xylose,72h

Kim et al., 2000: 1.35 L anaerobic fermentor, 30oC, pH 6, ~ 10% inoculum, 50 g/L xylose

Delgenes et al., 1996: 100 mL shake flasks, 30^o^C, initial pH 5.6, 3% inoculum, 20 g/L xylose and 20 g/L glucose, 24h

Chen et al., 2012: 20 mL test tube, 30^o^C, initial pH 6, anaerobic, 0.01 inoculum OD_600_, 50 g/L glucose, 24h

This study: 300 uL Bioscreen C microplate, 30^o^C, pH 5.8, .05 inoculum OD_600_, 20 g/L glucose (24 h) or xylose (48 h)

Joachimsthal et al., 1998: 1L anaerobic fermentors, 30^o^C, pH 5.0, 0.15 g/L cell inoculum, sodium acetic acid, 100 g/L,

Mohagheghi et al., 2004: 500 mL fermentors, 30^o^C, pH 6 with KOH, 0.2 inoculum OD_600_, 40 g/L glucose and 40 g/L xylose,120h

Fein et al., 1984: 10 mL, 30^o^C, initial pH 5.5, OD, 50 g/L glucose; used broth dilution method for MIC calculation
